# Quality of life after emergency laparotomy: a systematic review

**DOI:** 10.1186/s12893-024-02337-y

**Published:** 2024-02-26

**Authors:** Esha Khanderia, Ravi Aggarwal, George Bouras, Vanash Patel

**Affiliations:** 1 Watford General Hospital, West Hertfordshire Teaching Hospitals NHS Trust, Watford, UK; 2 Department of Surgery & Cancer, Imperial College London, London, UK; 3 University Hospitals of North Midlands NHS Trust, Stoke-on-Trent, UK

**Keywords:** Quality of Life, Emergency Laparotomy, Care of the Elderly, QOL, Survivorship, Abdominal Surgery

## Abstract

**Background:**

Emergency laparotomy is a commonly performed surgical procedure that has higher post-operative morbidity and mortality than elective surgery. Previous research has identified that patients valued postoperative quality of life (QoL) more than the risk of mortality when deciding to undergo emergency surgery. Current pre-operative scoring and risk stratification systems for emergency laparotomy do not account for or provide prediction tools for post-operative QoL. This study aims to systematically review previous literature to determine post-operative QoL in patients who undergo emergency laparotomy.

**Methods:**

A literature search was undertaken in Medline, EMBASE and the Cochrane Library to identify studies measuring post-operative QoL in patients who have had emergency laparotomy up to 29th April 2023. Mean QoL scores from the studies included were combined to calculate the average effect of emergency laparotomy on QoL. The primary outcome of the review was postoperative QoL after emergency laparotomy when compared with a comparator group. Secondary outcomes included the quality of included studies.

**Results:**

Ten studies in the literature assessing the QoL of patients after emergency laparotomy were identified. Three studies showed that patients had improved QoL and seven showed worse QoL following emergency laparotomy. Length of time for QoL to return to baseline varied ranged from 3 to 12 months post-operatively. Length of hospital stay was identified as an independent risk factor for poorer QoL post-surgery.

**Conclusions:**

Outcome reporting for patients who undergo emergency laparotomy should be expanded further to include QoL. Further work is required to investigate this and elicit factors that can improve QoL post-operatively.

## Introduction

Emergency laparotomy is a commonly performed surgical procedure that has higher post-operative morbidity and mortality than elective surgery [1].

The age of patients should not be considered in isolation, because the risk of morbidity and mortality to patients depends on many pre-operative, intra-operative and post-operative factors [2, 3]. Previous research has identified that patients valued postoperative quality of life (QoL) more than the risk of mortality when deciding to undergo emergency surgery [4]. It was also shown that patients and clinicians had different views on what defined a positive outcome after emergency laparotomy. Current pre-operative scoring and risk stratification systems for emergency laparotomy such as the National Emergency Laparotomy Audit (NELA) score or Portsmouth Physiological and Operative Severity Score for the 2 numeration of Mortality and morbidity (P-POSSUM) do not take into account or provide prediction tools for post-operative QoL [5].

The 30-day mortality for emergency laparotomy in the United Kingdom is 10.6% but in patients over the age of 70, this is almost double at 20% [6]. In older patients, there is a higher burden of post-operative complications and more complex social and care challenges resulting in longer lengths of stay [7, 8]. Clinicians have primarily led outcomes reporting with a focus on mortality and length of stay [4], however, there is little focus on QoL in this cohort of patients [8]. Therefore, the aim of this study is to systematically review previous literature to determine the post-operative QoL in patients who undergo emergency laparotomy.

## Methods

Patients who underwent laparotomy for emergency general surgery conditions were identified to assess their QoL after the surgical procedure. The primary outcome of the review was postoperative QoL after emergency laparotomy when compared with a comparator group. Secondary outcomes included the quality of included studies. The study is registered with PROSPERO, CRD42023434841.

### Literature search

The study was conducted in accordance with the guidelines for the ‘Preferred Reporting Items for Systematic Reviews and Meta-Analyses (PRISMA)‘ [9]. Electronic bibliographic searches were conducted in Medline, EMBASE and the Cochrane Library combining MESH and all-field search terms for “quality of life” OR “survivorship” AND “emergency laparotomy”. Studies from 2000 onwards were included to ensure that the practice reflected the current surgical management of emergency conditions. Further studies were identified through manual searches of bibliographies and citations. The final search was completed on 29th April 2023.

### Inclusion and exclusion criteria

Studies were included if they measured post-operative QoL in patients who had undergone emergency laparotomy for a general or gastrointestinal surgical condition and had a comparator group. For the purposes of this analysis, emergency laparotomy was defined as an open major abdominal surgery and excluded laparoscopy, gynaecological and vascular procedures. Articles using generic and disease-specific QoL instruments were included. Exclusion criteria were studies that did not include post-operative QoL as an outcome measure, studies that evaluated tools to measure QoL but did not specifically assess the QoL in patients undergoing emergency laparotomy, studies in children and review articles.

### Study selection

Two investigators (E.K. and R.A.) independently screened titles and abstracts and selected all relevant citations for full-text review. Disagreement regarding study inclusion was resolved by discussion with the third investigator (V.P.). The full texts of relevant articles were reviewed, and corresponding authors were contacted for other sources of data if applicable.

### Study quality

The quality of the studies was measured using a quality assessment score adopted from previous reviews of QoL studies [10–12]. One point was assigned for each of the 11 items in the assessment criteria. A score of higher than 8 indicated a high-quality study, 5 to 7 was a moderate quality study and 4 or lower was a poor-quality study.

### Data Collection & Analysis

Data on first author, year of publication, study design, number of patients, QoL instruments, QoL components, response rates, follow-up, mean QoL scores in post-operative and comparator group were collected.

Reported QoL scores derived were from the mean difference in postoperative QoL scores between the post-operative group and the comparator groups. Mean QoL scores from different studies were combined to calculate the average effect of emergency laparotomy on QoL. As per a previous QoL review [10], the different QoL measurement tools were scaled down to a 0 to 1 score by dividing the maximum for the QoL tool used.

## Results

### Search results

**Fig. 1:**
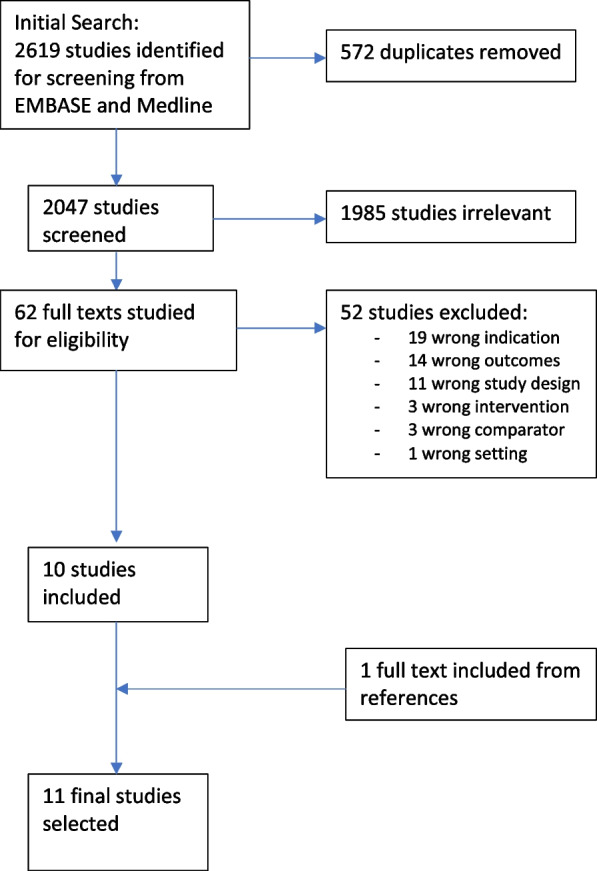
Study selection PRISMA flow chart

Our search identified 2619 abstracts, of which 2047 were screened after duplicates were removed. Of these, 1985 did not fulfil inclusion criteria based on title and abstract. Full-text review was performed for the remaining 62 papers. From these, 10 studies were selected, and one further study was identified from bibliographic searches. Eleven studies were included in the final review (see Fig. 1), producing a pooled data set of 1542 patients with an average age of 61.2 years. These 11 studies varied in quality (Table 1) with quality scores ranging from 5 to 9. Four studies were of high quality and seven were of moderate quality. The mean quality score for the studies included was 7.

**Table 1:** Study Quality Assessment

	Witte et al. 2022 [22]	Alder et al. 2021 [8]	Purcell et al. 2021 [13]	Saunders et al. 2021 [14]	Tolstrup et al. 2019 [15]	Kwong et al. 2018 [19]	Li et al. 2017 [16]	Jeppesen et al. 2016 [20]	Boer et al. 2007 [17]	Joneja et al. 2004 [18]	Scheingraber et al. 2002 [21]
QOL is compared between groups	1	1	1	1	1	1	1	1	1	1	1
The point of follow up is defined prospectively	0	0	1	1	0	1	1	0	1	1	0
Response rates > 75%	0	0	0	0	0	0	1	1	1	1	1
Characteristics of non-responders given	1	0	1	1	1	1	1	0	1	0	0
Validated QOL instrument	1	1	1	1	1	1	0	1	1	1	1
Mean values reported	0	1	1	1	1	1	0	1	0	1	0
Consent/ethical approval is described	1	0	1	1	1	1	1	1	0	0	1
Pre and post-operative QOL is measured	0	1	1	1	0	1	1	0	0	1	0
Missing data imputation stated	0	0	1	0	1	1	0	0	0	0	0
Accounts for confounding factors	0	0	0	0	1	0	0	1	1	0	0
Selection criteria are formulated	1	1	1	1	1	1	1	1	1	1	1
**TOTAL SCORE (out of 11)**	**5**	**5**	**9**	**8**	**8**	**9**	**7**	**7**	**7**	**7**	**5**

### Study design

The studies included consist of six prospective [13–18] and five retrospective [8, 19–22] studies. The mean follow-up for the prospective studies was 13.7 months and 14.8 for the retrospective studies. Prospective studies had a higher mean quality score than retrospective studies (7.7 vs 6.3 respectively). Five studies matched post-operative QoL to the pre-operative QoL of the same patient cohort [13, 14, 16, 18, 19], four studies compared the post-operative patients to a healthy reference population [9, 18, 22, 23] and two studies compared those with post-operative chronic pain to patients with no post-operative pain [15, 20] (Table 2).

**Table 2:** Summary of Studies Included in Literature Review

Author and year	Method	Average age (years)	Preoperative QOL	N (eligible Patients)	Follow up (months)	Mortality	Postoperative QOL	QOL measurement tool	Baseline/Comparator Population	Components with significant differences	Questionnaire Response Rate (%)
Witte et al. 2022 [22]	Retrospective	71	No	64	49.4	60.9% in hospital	Yes	EQ 5D	Reference population	Not available	19
Alder et al. 2021 [8]	Retrospective	79	Yes	153	19	35.3% all-cause mortality at 19 months post-operatively	Yes	SF-36	General population	Physical functioning reduced Energy and fatigue worse Social functioning decreased	64.7
Purcell et al. 2021 [13]	Prospective	39	Yes	117	1	2.6% in hospital	Yes	PROMIS-25	Preoperative status (questionnaire administered up to post-op day 1)	Fatigue increased Mobility improved Pain worsened	65.8
Saunders et al. 2021 [14]	Prospective	65.5	Yes	129	12	Not reported	Yes	EQ 5D WHODAS	Preoperative status (questionnaire administered at post-op day 5)	Not available	54
Tolstrup et al. 2019 [15]	Prospective	69	No	605	60	Not reported	Yes	GIQLI	Patients with no chronic post-operative pain	Core symptoms worse Physical items worse Psychological items worse Social function worse Disease specific items worse	73
Kwong et al. 2018 [19]	Retrospective	65	Yes	255	3	Retrospective using same patient group, only alive patients included in study	Yes	EQ 5D3L GIQLI	Preoperative status (retrospective questionnaire regarding preadmission health status)	Improved symptoms Reduced social functioning	74.1
Li et al. 2017 [16]	Prospective	65.3	Yes	50	3	Not reported	Yes	Unspecified	Preoperative status (timing of questionnaire not specified)	Not available	100
Jeppesen et al. 2016 [20]	Retrospective	61	No	110	37	Retrospective, only alive patients included in study	Yes	GIQLI	Patients with no chronic post-operative pain	Symptoms worse Emotions worse Medical treatment increased	82
Boer et al. 2007 [17]	Retrospective	63	No	155	6	Retrospective, only alive patients selected for inclusion	Yes	EQ 5D EQVAS	Healthy reference population	Mobility worse Self-care reduced Daily activity reduced Pain worse Mood worse	84
Joneja et al. 2004 [18]	Prospective	41.5	Yes	51	6	Not reported	Yes	GIQLI	Preoperative status (questionnaire completed before surgery)	Improved disease specific symptoms Psychological symptoms improved Physical and social factors improved	84.3
Scheingraber et al. 2002 [21]	Retrospective	54	No	60	unknown	46% in hospital, 10% at home (unspecified time post-operatively)	Yes	SF-36	Matched pair in normal population	Physical function worse Physical role worse Mental health improved	97

### QoL tools

The QoL tools used in the included studies were Short Form-36 (SF-36) (2 studies), EuroQol 5 Dimensonal (EQ-5D) (4 studies), Gastrointestinal quality of life index (GIQLI) (4 studies) and World Health Organisation Disability Assessment Score (WHODAS), EuroQol Visual analogue scales (EQVAS) and Patient Reported Outcomes Measurement System 25 (PROMIS-25) one study each. These scores are all validated scoring systems for assessing QoL. The type of QoL tool used was unspecified in one study.

### Pre- and post-operative QoL comparison

In total five studies compared post-operative QoL with a pre-operative baseline. Of these, three studies provided numerical QoL comparison scores and two provided graphical representations of the data from which exact values were not obtainable (Table 3). One study showed that the pre-operative QoL was better than the post-operative QoL [14]. However, three studies identified that post-operative QoL was better than pre-operative QoL [16, 18, 19]. The other study described the individual parameters of their QoL assessment tool and showed that emotional domains, fatigue and pain worsened post-operatively, however mobility marginally improved [13]. No studies performed multivariate analyses to try to identify predictive factors for change in post-operative QoL.

**Table 3:** Overall mean differences in post-operative QoL compared to comparator groups

Study	Baseline/Comparator Population	QoL instrument	Post-operative QoL score	Comparator Group QoL score	Mean Difference Post-op vs Comparator	*p* value	Post-operative QoL compared to comparator group	Questionnaire Response Rate (%)	Comments
Witte et al. 2022 [22]	Reference population	EQ 5D	–	–	–	–	Lower	19	Numerical scores not disclosed
Alder et al. 2021 [8]	General population	SF-36	0.529	0.591	−0.062	0.334	Lower	64.7	–
Purcell et al. 2021 [13]	Preoperative status (questionnaire administered up to post-op day 1)	PROMIS-25	–	–	–	–	Lower	65.8	Overall QoL scores not disclosed
Saunders et al. 2021 [14]	Preoperative status (questionnaire administered at post-op day 5)	EQ 5D	–	–	–	–	Lower	54	Numerical scores not disclosed
		WHODAS 2.0	0.042	0.063	−0.021	–	Lower		–
Tolstrup et al. 2019 [15]	Patients with no chronic post-operative pain	GIQLI (chronic pain)	0.681	0.847	−0.167	0.01	Lower	73	Comparison score is patients without chronic pain
Kwong et al. 2018 [19]	Preoperative status (retrospective questionnaire regarding preadmission health status)	EQ 5D	0.640	0.580	0.060	0.06	Higher	74.1	–
		GIQLI	0.680	0.648	0.032	0.048	Higher		–
Li et al. 2017 [16]	Preoperative status (timing of questionnaire not specified)	Unspecified	–	–	–	–	Higher	100	Numerical scores not disclosed
Jeppesen et al. 2016 [20]	Patients with no chronic post-operative pain	GIQLI	0.861	–	–	–	Lower	82	–
		GIQLI (chronic pain)	0.757	0.882	−0.125	< 0.001	Lower		Comparison score is patients without chronic pain
		AAS	–	–	–	–	Lower		Numerical scores not disclosed
Boer et al. 2007 [17]	Healthy reference population	EQ 5D	–	–	–	< 0.001	Lower	84	Numerical scores not disclosed
		EQVAS	–	–	–	–	Lower		Numerical scores not disclosed
Joneja et al. 2004 [18]	Preoperative status (questionnaire completed before surgery)	GIQLI	0.917	0.616	0.301	< 0.001	Higher	84.3	3 months post op
		GIQLI	0.869	0.616	0.253	< 0.001	Higher		6 months post op
Scheingraber et al. 2002 [21]	Matched pair in normal population	SF-36	–	–	–	–	Lower	97	Numerical scores not disclosed

### Variations in QoL during the post-operative period

Four prospective studies collected data at predefined time frames along the post-operative journey to determine the changes in QoL at these points. Purcell et al. collected QoL questionnaires preoperatively and on day 7 and day 30 post-operatively. This study showed that compared to their pre-operative status, patients had increased anxiety, pain, depression and fatigue on post-operative day 7 and this did not significantly change by day 30 [13]. Saunders et al. collated QoL data at 1, 3, 6 and 12 months post-operatively and showed that whilst QoL decreases in the immediate post-operative period of one to 3 months, it seemed to return back to baseline by 6–12 months [14]. Li et al. illustrated that QoL at 1 month and 3 months post laparotomy was better than the baseline [16], a finding also shown by Joneja et al. at 3 and 6 months [18].

### Post-operative QoL compared with a normal population

Four studies compared the post-operative QoL with a normal healthy reference population [9, 18, 22, 23]. One study provided numerical values and the other two studies displayed their results in graphical form. These studies all demonstrated that post-operative QoL was impaired for patients in comparison to their reference population. Scheingraber et al. showed that these domains all improved from 1 year post-operatively, however the physical and emotional parameters were still not at baseline by this point [21].

Multivariate analyses were performed in one study [17]. In this study, the authors adjusted for confounders including age, sex, co-morbidities, length of stay and presence of enterostomy. Length of stay was identified as the only independent factor that was predictive for worse post-operative QoL on the EQ-VAS scale. Increasing length of stay was also identified as an independent risk factor for worsening QoL in all dimensions on the EQ-5D scale.

### Post-operative QoL in patients with and without chronic pain

Two studies compared post-operative gastrointestinal QoL in patients in patients who developed chronic abdominal pain following emergency laparotomy and those who did not [15, 20]. Both studies show that patients who developed chronic pain had a reduced gastrointestinal QoL in comparison to those who did not develop chronic pain and these scores were found to be statistically significant.

Multivariate analyses were performed in one study [15] which identified that acute post-surgical pain and age were independent predictors for developing chronic post-surgical pain which in turn leads to a reduced QoL post laparotomy.

### Factors affecting QoL

Four studies demonstrated significant worsening of physical function, mobility or self-care post-operatively [8, 15, 17, 21]. In addition, three studies reported worse social function [8, 15, 19], three reported worse psychological items or emotions or mood [15, 17, 20], two reported worse energy levels or increased fatigue [8, 13] and two reported worse pain post operatively [13, 17]. One study showed that the length of bowel resected correlated to impairment of QoL [22]. Only one study from those included in this review reported significant improvement in all domains of QoL measured after emergency laparotomy [18].

## Discussion

QoL after emergency laparotomy is not routinely considered or measured when making decisions about active treatment for emergency surgical conditions [23]. The key findings of this systematic review of the literature show that there are very few studies assessing the QoL of patients after emergency laparotomy. The studies identified had significant variation in their methods, QoL instruments utilised, comparator groups, outcomes reported and used a combination of numerical and graphical scores. Of the 11 studies included, six enabled assessments of QoL post-operatively in relation to comparator groups through numerical scores, four through graphical representations and one through non-cumulative numerical scores in the manuscript. This heterogeneity in study design and outcomes reported made interpretation and evaluation difficult.

Post-operative QoL was found to be lower in 8 of the 11 studies included. Physical, social and psychological QoL were found to be commonly affected post-operatively. Three of the 11 studies showed improved post-operative QoL [16, 18, 19]. The younger average age of patients in the studies that showed improved QoL may account for better physical health and function, higher levels of physiological reserve and faster recovery from major emergency surgery which led to an improved post-operative QoL. Our review has shown that most patients have a reduced QoL for at least 3 months post-operatively caused by lower physical functioning, social functioning and energy levels than either the normal population or their pre-operative status. The average age of patients in our study was 61.2 years; in older patients with more co-morbidities and lower baseline level of function, QoL may be impaired for longer than 3 months post-operatively due to the length of time taken to recover from complications or prolonged hospital stay and deconditioning. Old age increases the risk of longer length of stay, complications and likelihood of ICU admission [23]. Alder et al. showed that 25.5% of septuagenarians undergoing emergency laparotomy were discharged to rehabilitation, intermediate care, residential homes, nursing homes, hospices or palliative care hospitals and reported an inpatient mortality rate of 13.7% [8]. Research comparing the effects of emergency laparotomy on QoL in a variety of age groups should be undertaken to establish whether age has a true impact on QoL.

The strongest predictor for lower QoL was found to be the length of hospitalisation [15, 17]. This suggests that pre-existing comorbidities and post-surgical complications that contribute to a longer length of stay, prolonged recovery and affect physical health may affect QoL, especially in the elderly [14, 15]. Therefore, more resources should be allocated to minimising hospital stay, utilising enhanced recovery techniques such as consideration of minimally invasive techniques where possible, intensive physiotherapy to avoid deconditioning, early mobilisation, adequate nutrition and early discharge planning.

Five studies [13, 14, 16, 18, 19] compared post-operative QoL to the pre-operative QoL in the same population. There is considerable variation in the timing of when the questionnaires were administered pre-operatively, ranging from pre-operatively to up to day 5 post-operatively. From the literature, in four of these studies, it is unclear if the authors have asked the patients to provide answers regarding their pre-operative QoL in relation to their pre-morbid health, or their health immediately pre-operatively. This may introduce recall bias into their responses as in the immediate pre-operative period, patients will perceive their QoL as poor due to illness and trying to recall their pre-morbid QoL at this time is also challenging. However, as post-operative QoL in this study is generally worse than pre-operative QoL, patients were likely reporting their pre-morbid health status when responding to the questionnaires.

There is a variability in response rates to the QoL surveys undertaken across all the studies. There appears to be no correlation between the response rates to the questionnaires and reported QoL as both studies with high and low QoL have variable response rates. Therefore, it is difficult to determine whether those patients with a lower QoL are more likely to complete the questionnaire to report on their ill health or whether patients with a higher QoL are keen to demonstrate their good health by responding. It is unclear why there is such variability between response rates across studies – attrition in long-term studies is a known challenge, however, there appears to be a reduced number of responses in certain studies which have a short follow-up duration of 1(ref Purcell et al) and 3 (ref Kwong et al) months where this effect would not necessarily be expected. It is important to note that there is a mortality associated with emergency laparotomy and therefore some of the non-responders may not have survived the follow-up term to report on their QoL. This however does not alter the findings of this study as the aim is to look at QoL in survivors of emergency laparotomy.

Emergency laparotomies are primarily performed as lifesaving procedures in critically unwell patients. Two studies looked at patients undergoing emergency laparotomy for peptic ulcer perforations [16, 18]. It is possible that the nature of the operation and population demographic who develop this condition benefit from improved QoL post-operatively due to treatment of their underlying disease pathology with surgery. Post-operative QoL may be dependent on the nature of the diagnosis that required laparotomy, with some operations such as a Hartmann’s procedure having life-altering impacts on QoL including managing a stoma [4, 17]. Underlying malignancy is also a factor that should be taken into consideration. One study (ref Scheingraber et al) looked at patients who underwent emergency laparotomy and had an underlying malignancy. They found that this cohort of patients had significantly impaired physical function within the first year after surgery, however, they generally recovered physically after this time although they reported emotional difficulties beyond a year. Cancer is an important factor and influences QoL in many domains, within the timeframe of a year post-operatively, patients may be undergoing adjuvant treatments in addition to their recovery which could further impact and confound their QoL reporting. It is crucial therefore that indications for emergency laparotomy are clearly reported in studies as these have an impact on prognosis, complications and QoL.

One study randomly assigned patients to receive either laparoscopy to laparotomy for the management of peptic ulcer disease [16]. Their study found that QoL was the same at baseline for patients undergoing both laparotomy and laparoscopy, however, at both one and 3 months post-operatively, the QoL for patients who had undergone laparoscopy was higher than those with laparotomy. This may be due to less pain, fewer complications and faster post-operative recovery with less invasive surgery. This demonstrates that the indication for surgery and method of operation plays a large factor in post-operative QoL. Furthermore, two studies have shown that patients with chronic pain post-emergency laparotomy have a significantly worse QoL than their counterparts with no chronic pain [15, 20]. Whilst this may be an expected finding, age and acute post-surgical pain were found to be predictors of chronic pain. This alludes to the importance of adequate post-operative analgesia including the use of epidurals or other invasive analgesic methods, especially in the elderly.

**Fig. 2:**
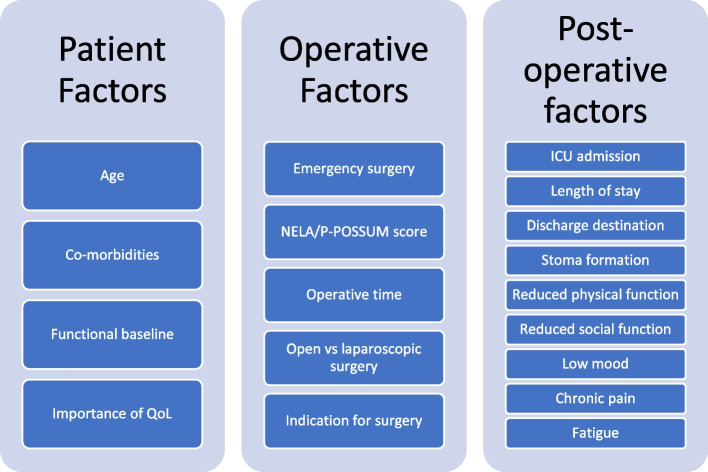
Factors affecting Quality of Life in patients undergoing emergency laparotomy

Many studies have assessed how QoL is affected in patients undergoing elective surgery and other emergency procedures [10], however, our review suggests that there is little research specifically focusing on QoL following emergency laparotomy. QoL is an essential factor to consider when planning care for these patients as their QoL may be normal prior to undergoing lifesaving emergency surgery. Therefore, it is crucial to identify if this is likely to change post-operatively and to ascertain the contributing factors (see Fig. 2). Emergency laparotomies are typically performed in an acute setting for an emergency surgical condition and therefore unlike other major abdominal operations, there is often no possibility of prehabilitation, optimisation of underlying pathology or deferral to watch and wait strategies. As patients are more unwell and require operations in a time-critical manner, the options for patients to decline or defer an operation due to risk are limited as the procedure may be lifesaving, altering the risk-benefit ratio. In order to improve QoL in patients undergoing emergency laparotomy surgeons must take a holistic approach to management at every stage of surgical care. This should include careful patient selection with open discussions regarding post-operative morbidity and QoL utilising available validated predictor scores to aid decision making [24]. The use of less invasive operative techniques such as laparoscopy [16] and further investment into post-operative recovery care including pain management, nutrition, physiotherapy and mobilisation, management of post-operative change in bowel habit, reducing stoma formation, recognition of post-operative complications and timely intervention are crucial areas of decision making in the peri-operative period and contribute to post operative QoL. Furthermore, in the elderly, involvement of Care of the Elderly physicians to provide a holistic approach to management is essential in managing this complex cohort of patients with multiple co-morbidities [25].

This review has highlighted the feasibility of collecting QoL data on patients undergoing emergency surgery and the need to consider the patient’s perspective regarding the impact of emergency laparotomy. Further work is required to expand the use of appropriate QoL frameworks routinely in patients undergoing emergency laparotomy and standardisation of methodologies for future QoL studies is needed. The NELA audit questionnaire, which is routinely conducted for the majority of patients undergoing emergency laparotomy in the United Kingdom, offers a potential avenue for the implementation and collection of QoL data from patients at scale. An emergency surgery-specific QoL questionnaire could be added to the NELA audit questionnaire at different time points to routinely collect and analyse this data in order to inform clinical practice and improve patient care. This could involve identifying areas where patient outcomes can be improved or highlighting successful interventions that improve quality of life outcomes.

### Limitations

There were several limitations with this review that should be accounted for. Although the literature search was comprehensive and specific inclusion and exclusion criteria were adhered to, it is possible that some studies may have been missed that should have been included. The studies included varied in the QoL instruments used to measure QoL in the post-operative and comparator populations. This led to heterogenous data and inconsistency in reporting results with numerical or graphical scores. In addition, the comparator population varied between studies, as did the length of post-operative follow-up, and the indications for emergency laparotomy. Furthermore, the prospective studies were of higher quality than the retrospective studies included which may affect the analysis. Some QoL instruments had very short-term follow-up of 30 days and these results are likely to be skewed due to lower scores in the immediate post operative period compared to longer term follow-up.

## Conclusions

Outcome reporting for patients who undergo emergency laparotomy should be expanded further to include validated measures of QoL. The studies included in this review have demonstrated the feasibility of collecting patient-reported outcomes in an emergency setting. The findings of this review inform the design of future studies that can identify where improvements can be made and resources allocated to this important group of patients. Therefore, further work is required to investigate how the routine collection of QoL data can be expanded for all patients undergoing emergency laparotomy and to elicit factors that can improve QoL post-operatively such as patient selection or the use of less invasive operative techniques where possible. Furthermore, there is a need for further research to evaluate whether age, specific operations and particular co-morbidities are accountable for adversely affecting QoL after emergency laparotomy.

## Data Availability

All data is available in the manuscript.
